# Paracentral acute middle maculopathy with cerebral infarction caused by carotid dissection: a case report

**DOI:** 10.3389/fneur.2025.1698882

**Published:** 2025-10-30

**Authors:** Huiqiong Tang, Lu Liu, Le Cao, Junfeng Liu, Bo Wu

**Affiliations:** ^1^Department of Neurology, Huize County People’s Hospital, Qujing, Yunnan, China; ^2^Department of Neurology, West China Hospital, Sichuan University, Chengdu, China; ^3^Health Management Center and General Practice Medical Center, West China Hospital, Sichuan University, Chengdu, China

**Keywords:** paracentral acute middle maculopathy, cerebral infarction, carotid dissection, optical coherence tomography, ischemic cascade

## Abstract

**Introduction:**

Paracentral acute middle maculopathy (PAMM) is related to retinal capillary ischemia, occurring independently or secondary to systemic diseases. We report an unusual case of PAMM with cerebral infarction caused by carotid dissection without systemic risk factors.

**Case description:**

A 55-year-old man presented with acute painless left visual loss and partial sensory aphasia, followed by right-sided weakness. Best-corrected visual acuity (BCVA) was 0.04 in the left eye. Optical coherence tomography (OCT) showed hyperreflective lesions at the OPL-INL junction; enface imaging revealed fern-like hyperreflective lesions in the inner nuclear layer (INL). OCT angiography (OCTA) demonstrated diffuse macular hypoperfusion. Brain MRI showed left basal ganglia infarction, and angiography confirmed left internal carotid artery (ICA) dissection and near occlusion. After carotid stenting and dual-antiplatelet therapy, BCVA improved to 0.8 at 3 months with OCTA-confirmed retinal reperfusion.

**Conclusion:**

This is a report that links PAMM and cerebral infarction to carotid dissection, suggesting that PAMM should prompt urgent stroke and carotid workup in appropriate clinical context.

## Introduction

Paracentral acute middle maculopathy (PAMM) is a recognized optical coherence tomography (OCT) feature marked by hyperreflective, band-like lesions localized to the inner nuclear layer (INL), typically observed in cases of retinal capillary ischemia (1). These ischemic changes frequently occur in anatomically vulnerable areas, such as deeper layers of the retina—border regions between the outer plexiform layer (OPL) and INL (2). PAMM may occur as an isolated condition or secondary to retinal vascular disorders or systemic diseases. While conventional fundus examination often reveals only subtle retinal changes (frequently neglected), OCT imaging enhances detection by revealing distinct morphological patterns. PAMM has been clinically associated with stroke and carotid artery diseases, and may serve as a critical indicator for urgent multidisciplinary stroke evaluation (3). Previous cases reported associations between PAMM and carotid artery atherosclerotic stenosis (1, 4). However, these patients had preexisting vascular risk factors (e.g., hypertension, diabetes, dyslipidemia), which are also associated with PAMM pathogenesis. Here, we share a unique case of PAMM and cerebral infarction caused by carotid dissection without any systemic diseases, highlighting the direct association between carotid pathology and PAMM.

## Case description

A 55-year-old man with no history of hypertension, diabetes, dyslipidemia, tobacco use, or any medications, presented with acute painless left eye vision loss and concurrent difficulty understanding others’ speech, and irrelevant responses, that started 3 days prior. The patient denied any neck trauma, or accompanying headaches, joint pain, or facial pain. Right-side facial/extremity muscle weakness and dysarthria were not present initially but developed 2 days after vision loss.

On emergency presentation, best-corrected visual acuity (BCVA, decimal) was 1.0 (right eye) and 0.04 (left eye). Extraocular motility was normal, while a relative afferent pupillary defect of left eye was found. Physical examination revealed partially irrelevant answers, decline in calculation and recent memory, left deviation of the mouth corner, weakness in right cheek puffing, decreased muscle strength in the right upper extremity while Horner’s syndrome was not found.

**Figure 1:**
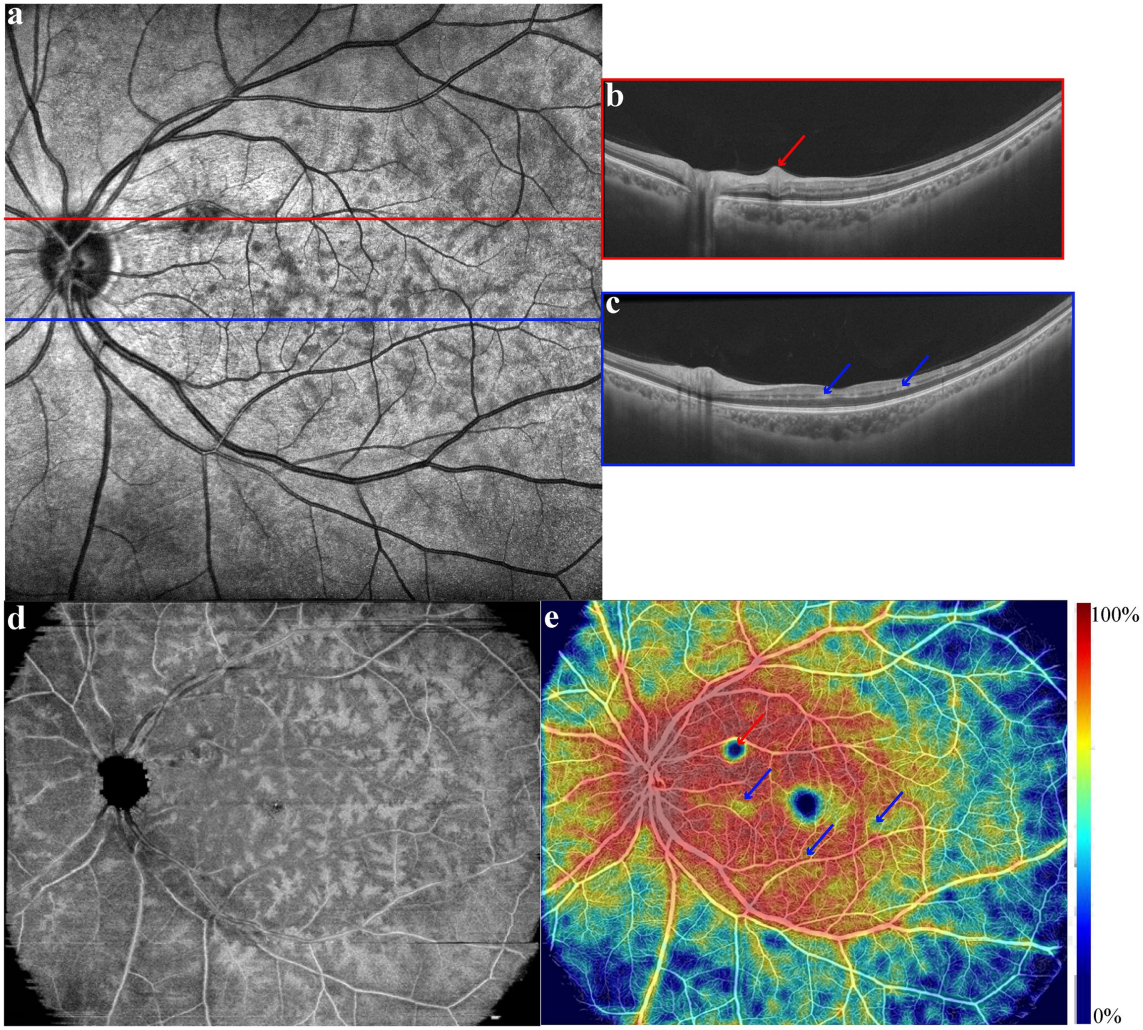
**(a)** One cotton-wool spot and multiple dark patches around the macula in infrared fundus images of left eye; **(b)** isolated hyperreflective lesions at retinal nerve fiber layer of red line slice; **(c)** hyperreflective lesions in the junction of the outer plexus layer and the inner nuclear layer of blue line slice; **(d)** fern-like hyperreflective lesions around retinal vessels in enface image of the inner nuclear layer; **(e)** diffuse low-vessel-density areas of retinal vessels on optical coherence tomography angiography; the color scale showed the vessel density (%) of retinal vessels.

Both eyes had normal anterior segment examination. Fundus examination of the right eye was normal, while left eye showed one macular cotton-wool spot, normal vessels and optic disc, and no hemorrhages. Infrared fundus images showed multiple macular dark patches (Figure 1a). Swept-source OCT (VG200S; SVision Imaging; version 2.1.016) of the right eye was normal while left eye demonstrated an isolated hyperreflective lesion of the retinal fiber layer corresponding to the cotton-wool spot (Figure 1b) and band-like hyperreflective lesions at the OPL-INL junction (Figure 1c) on OCT slices. Enface image of INL demonstrated fern-like hyperreflective lesions around retinal vessels (Figure 1d). Simultaneous enface OCT angiography (OCTA) revealed diffuse low-vessel-density areas of retinal vessels (Figure 1e) around the left macula.

**Figure 2:**
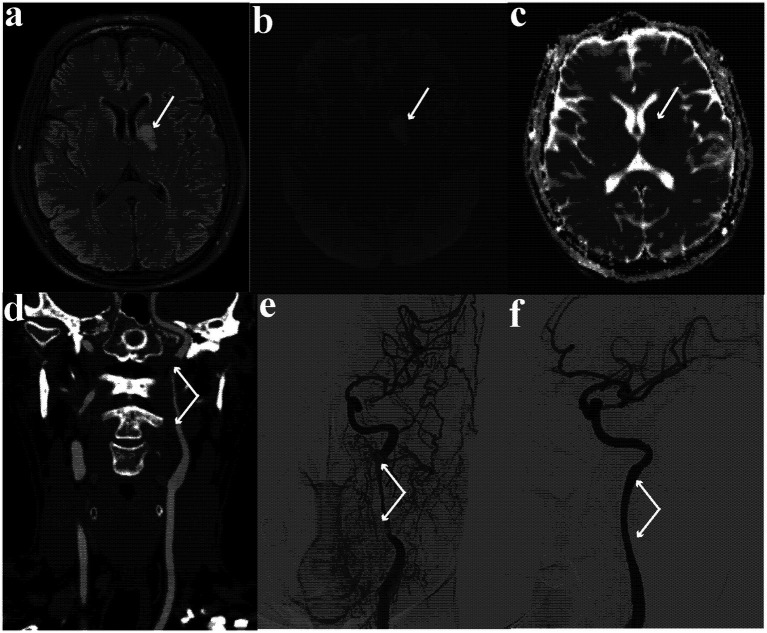
**(a)** Hyperintense oval lesion at left basal ganglia and inner capsule on fluid-attenuated inversion recovery image; **(b)** hyperintense oval lesion on diffusion-weighted imaging; **(c)** reduced apparent diffusion coefficient at the lesion; **(d)** near occlusion at the upper cervical segment of internal carotid artery and wave-like tunica intima of proximal carotid artery on computed tomography angiogram; **(e)** carotid dissection with severe stenosis on digital subtraction angiography; **(f)** repaired internal carotid artery and patent left ICA lumen.

Brain magnetic resonance imaging showed an oval hyperintensity lesion at the left basal ganglia and inner capsule (in the territory of the left internal carotid artery) on fluid-attenuated inversion recovery (Figure 2a) and diffusion-weighted imaging (Figure 2b), and reduced apparent diffusion coefficient at the lesion (Figure 2c). Computed tomography angiography showed near-occlusion of the upper cervical segment of the left internal carotid artery (ICA) and wave-like tunica intima of proximal ICA (Figure 2d), confirmed as carotid dissection with severe stenosis on digital subtraction angiography (Figure 2e).

His workup included a normal Holter electrocardiogram and echocardiogram, normal complete blood count examination, normal-level low-density lipoprotein, erythrocyte sedimentation rate and hemoglobin A1c, with normal blood pressure (119/81 mm Hg). The findings on ophthalmic imaging (PAMM left eye) and neurologic imaging (cerebral infarction and carotid dissection) supported the clinical diagnosis of left side deep retinal ischemia and ischemic cerebral stroke, likely from spontaneous left inner carotid dissection.

The patient received dual-antiplatelet therapy (aspirin 100 mg and clopidogrel 75 mg, 6 months in plan) and underwent left cervical ICA stenting (Figure 2f) five days later. One week later, extremity weakness and dysarthria lessened drastically, with left eye BCVA recovering to 0.3, while aphasia remained.

**Figure 3:**
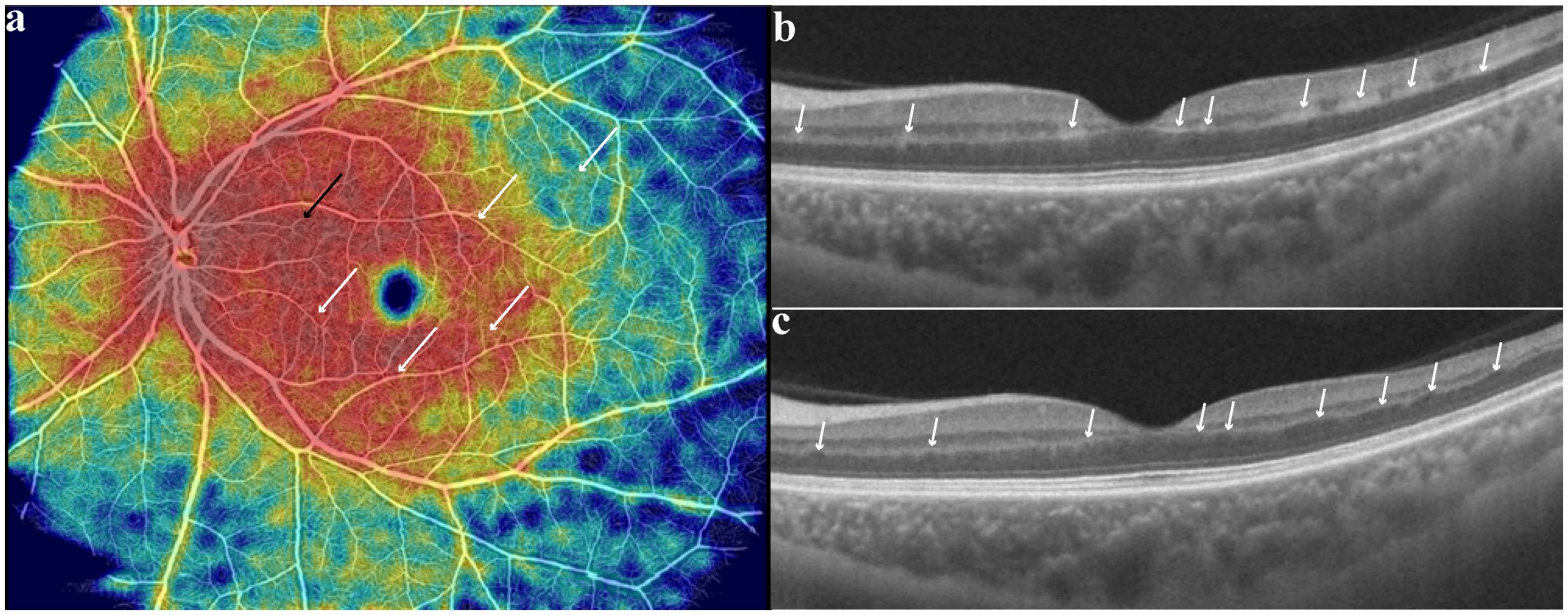
**(a)** Reperfusion of retinal vessels in previous low-vessel-density areas; **(b,c)** colocalized focal atrophy of the inner nuclear layer with a compensatory upward expansion of the outer nuclear layer into the outer plexiform layer, at previous hyperreflective foci site.

At 3-month follow-up, left eye BCVA improved to 0.8 and only slight aphasia remained. Enface OCTA image showed reperfusion of inner retinal vessels in previous low-vessel-density areas (Figure 3a). The hyperreflective foci on OCT disappeared, with focal INL atrophy and compensatory ONL expansion into the OPL at every previous hyperreflective foci site (Figures 3b,c). Neuroradiology follow-up imaging showed atrophy of previous site of infarction and patent left ICA stent.

## Conclusion

We report an unusual case of PAMM and ischemic stroke secondary to carotid artery dissection. PAMM is linked to retinal capillary ischemia and frequently occurs in anatomically vulnerable areas (1, 2, 5). With the advent of OCTA, reduced vascular vessel density is reported in PAMM cases (6) but the etiology of PAMM remains unknown. Idiopathic cases, or with systemic/retinal vascular diseases were reported. Our case demonstrates PAMM arising from carotid dissection, with concurrent cerebral infarction, in the absence of systemic risk factors.

Carotid dissection is a relatively rare etiology of ischemic stroke but an important cause of stroke in younger patients (7). It can lead to intraluminal thrombus or stenosis/occlusion, causing ischemic stroke by artery-to-artery embolism or hemodynamic failure. As the ophthalmic artery originates from the ICA, carotid dissection may manifest with several ischemic ophthalmic symptoms [e.g., transient monocular blindness, central or branch retinal artery occlusion (8), and ocular ischemic syndrome (9)]. Our case expands this spectrum by linking PAMM and concurrent cerebral ischemia to carotid dissection.

While no standardized management approach currently exists for PAMM, observational studies suggest that many cases demonstrate spontaneous resolution within 3–6 months of onset (4). The perivenular fern-like PAMM pattern may occur early in the retinal ischemic cascade (10) and this particular PAMM pattern has better visual prognosis as long as it has not developed into diffuse globular PAMM or inner retinal ischemia (11). Our patient underwent urgent carotid imaging and treatment, and experienced rapid visual/neurological improvement after carotid revascularization and dual-antiplatelet therapy.

In conclusion, PAMM should prompt clinicians to include urgent carotid artery evaluation in diagnostic protocols, as retinal ischemia may precede or coincide with cerebral infarction. Further studies are needed to establish the association between PAMM, cerebral infarction and carotid dissection.

## Data Availability

The datasets presented in this article are not readily available because of ethical and privacy restrictions. Requests to access the datasets should be directed to the corresponding author.
